# Et Tu Brute?

**Published:** 1885-08

**Authors:** 


### ET TU BRUTE?

Dr. A. G. Clopton, of Jefferson, Texas, in one of the best extempore efforts we ever had the pleasure of listening to, delivered at the Convention of the Texas State Medical Association at Houston in April, in speaking to his resolution endorsing the action of the legislative committee, and referring to attacks on that committee from members, said:

"The open enemy, the committee was prepared to confront, but an anonymous attack, from members of the Association, is like the assassin's danger in the hands of a brother. The great Cæsar confronted, in defiance, the conspirators in that last tragic scene, ready to fight for life and ambition; but when he saw the dagger in the uplifted hand of Brutus, whom he loved, more mortified at the treachery of his friend than alarmed at the danger which threatened him, he submitted to die."

Referring to the letter in our CORRESPONDENCE department conveying the resolutions of the Dallas County Medical Society on the International Congress question, we would not be understood as characterizing such action as "treachery on the part of our friends" by any means; but we cannot resist the impulse to make the comparison to Brutus stabbing Cæsar; for Texas was outspoken on the subject, and her large delegation—the largest of any State present—numbering one hundred and twenty, *voted* to stand by the Association, endorsing its course in asserting the right to supervise the action of a committee of its own appointing, or to dissolve the committee if need be.

We do not know how Dallas was represented in that convention, we do not remember but one delegate present from Dallas, and we *know* the one, to whom we refer, voted with the delegation as above; and we know, too, that he is a member of the Dallas Medical and Surgical Society. We were not aware there was a dissenting voice in Texas, to the action of the American Medical Association, nor to the action of the committee at Chicago, and hence this communication strikes us with surprise, as a blow, coming from an unexpected quarter, even from a brother. It grieves us, too, to see that such sentiment exists, and we much fear that it is of *very recent date*; since the Chicago meeting, in fact, and that the dissatisfaction might be suspected by less liberal persons than we, of springing from motives not unlike those these gentlemen assign to the dissenters at New Orleans, who objected to the appointment of new code men, and men from the northeast exclusively. That is to say—the committee at Chicago, in remembering that Texas is represented in the American Medical Association by a large membership, and is one of the states in the American Union, were guilty, in a small way, of the sin of which the original committee was charged; for it is a singular coincidence that none of the Texas appointees to the International Congress are from the northern or northwestern part of the State.

However, be our own sentiments what they may on this subject, this Journal professes to be the exponent of medical thought and opinion in our own State, and is therefore open, as well to those who differ with us upon subjects on which we feel deeply, as to those who coincide with us. It is, therefore, as before stated, our duty to give all opinions a hearing. Were we to do otherwise, were we to close our columns to such expression of sentiment, as those to which we are now alluding, coming from a source of such eminent respectability — men whose opinions are entitled to deference — we should be recreant to the trust reposed in us, and unworthy the liberal patronage and support accorded us by the Texas profession at large. And right here we wish to say, that however outspoken we may be, the EDITORIAL department is responsible alone for *our* sentiments, *this Journal* is *not* the organ of any ring, clique, combination or sentiment, but it aims to voice the sentiment of the profession at large, and to be an exponent of rational medicine, as exemplified in the practice of its members.

As said, we are pained at this dissent. We have had our 'say' and do not now propose to go into a discussion of the subject anew, further than to say we supposed the support by the highest recognized authority on parliamentary law and usage, given to the American Medical Association in the course it took at New Orleans, would surely make that course of action satisfactory to the entire South and West. It seems Dallas, too, begs leave to differ with Mr. ex-Speaker Randall and N. S. Davis, as well as with the Association.

We can imagine we see the smile of derision and triumph play over the countenance of certain of those we have been giving "fits" to—those whom we must call "the opposition;" but we publish the resolutions, all the same. We are something of a philosopher, and would rather laugh at our own expense that not to laugh at all. We are not going to do like Cæsar, ether, give up the fight, and die; when we do, we will die fighting back; at the same time, having "expended so much gas" on this subject already, as our big contemporary of the *New York Journal* would say, we will do like that little boy did, who is said to have written out his prayer, and tacked it to the footpost of his bed. We will refer these Dallas gentlemen to the editorial in the Journal of the American Medical Association for July 25, and say "them's our sentiments." If *that* article does not *convince* the most ultra conservative man in Texas that the Association is *right*, nothing we could say would be of avail. No, because Dallas has "gone back on us," we will not give up the fight; our cause is just and *must* prevail."

